# Exposure to environmental pollutants and attention-deficit/hyperactivity disorder: an overview of systematic reviews and meta-analyses

**DOI:** 10.1007/s11356-023-30173-9

**Published:** 2023-10-12

**Authors:** Eleonora Rosi, Alessandro Crippa, Marco Pozzi, Stefano De Francesco, Mariachiara Fioravanti, Maddalena Mauri, Massimo Molteni, Luisa Morello, Luca Tosti, Francesca Metruccio, Emilio Clementi, Maria Nobile

**Affiliations:** 1 Child Psychopathology Unit, Scientific Institute, IRCCS Eugenio Medea, Via Don Luigi Monza 20, Bosisio Parini (LC), 23842 Lecco, Italy; 2 Scientific Institute IRCCS E. Medea, Bosisio Parini, Lecco, Italy; 3 Sigmund Freud University, Sigmund Freud University of Milan, 20143 Milan, Italy; 4 University of Milano-Bicocca, Milan, Italy; 5 Pharmacovigilance & Clinical Research Unit and International Centre for Pesticides & Health Risk Prevention, Department of Biomedical and Clinical Sciences, “Luigi Sacco” University Hospital, Università degli Studi di Milano, Milan, Italy

**Keywords:** Attention deficit/hyperactivity disorder, Environmental pollutants, Air pollution, Persistent organic pollutants, Heavy metals, Phthalates, Overview of systematic reviews

## Abstract

Although heritability estimates suggest a role for genetic components, environmental risk factors have been described as relevant in the etiology of attention deficit/hyperactivity disorder (ADHD). Several studies have investigated the role of toxicological pollution, i.e., air pollution, heavy metals, POPs, and phthalates. Clear evidence for association of ADHD and environmental factors has not been provided yet. To answer this, we have assessed all available systematic reviews and meta-analyses that focused on the association between pollutant exposure and either ADHD diagnosis or symptoms. More than 1800 studies were screened of which 14 found eligible. We found evidence of a significant role for some pollutants, in particular heavy metals and phthalates, in the increased risk of developing ADHD symptoms. However, at the current stage, data from existing literature also do not allow to weight the role of the different environmental pollutants. We also offer a critical examination of the reviews/meta-analyses and provide indications for future studies in this field. PROSPERO registration: CRD42022341496

**Supplementary Information:**

The online version contains supplementary material available at 10.1007/s11356-023-30173-9.

## Introduction

Attention deficit/hyperactivity disorder (ADHD) is a complex neurodevelopmental condition with a known persistence into adulthood. It is characterized by inattention, motor hyperactivity, and impulsivity that are inconsistent with age or developmental level (Thapar and Cooper 2016; APA 2013). ADHD is predominantly present in males with a male-to-female ratio of 4:1 in clinical studies and 2.4:1 in population studies (Leopold et al. 2019). ADHD prevalence is now reported between 2 and 7% of children and adolescents worldwide, with an average prevalence of approximately 5% (Posner et al. 2020; Sayal et al. 2018). Recent data from the USA show an ADHD rate of about 12.9% (95% confidence interval: 11.5, 14.4%) (Zgodic et al. 2023). The most recent report by the Centers for Disease Control and Prevention indicates 6.1 million American children (9.4%) between the ages of 2 and 17 years have ever been diagnosed with ADHD, with approximately half of them aged 12–17 years (CDC 2019; Cabral et al. 2020).

ADHD may manifest itself differentially in terms of behavior, etiology, and developmental trajectories; it may occur in comorbidity and respond variably to interventions (Luo et al. 2019). A wide set of risk factors likely contribute to this heterogeneity. Although there is no comprehensive epidemiological model of ADHD, there is shared understanding that ADHD etiology is influenced by genetic tracts and environmental cues (Cabral et al. 2020; Nigg et al. 2010; Thapar et al. 2013; Thapar and Cooper 2016). Regarding environmental cues, a recent umbrella review confirmed that maternal pre-pregnancy obesity and overweight; pre-eclampsia, hypertension, acetaminophen exposure, and smoking during pregnancy; and childhood atopic diseases were strongly associated with ADHD (Kim et al. 2020). Nevertheless, several studies over the last few decades have investigated specific types of environmental risk factors, i.e., toxicological/pollution risk factors (e.g., Cheslack-Postava et al. 2022; Forns et al. 2018). Exacerbation of ADHD symptoms in children or an increased risk of developing ADHD has been often associated with air pollution (Aghaei et al. 2019; Myhre et al. 2018), defined as a complex mixture of particulate matter (PM), gases, trace metals, and adsorbed organic contaminants originating mainly from human activities, such as combustion of fossil fuels in combustion engines (Myhre et al. 2018). Ambient air pollution is thought to be involved in the pathogenesis of ADHD through prenatal exposure to the child, especially passing from the mother’s blood to the fetus (Aghaei et al. 2019; Zhang et al. 2017). Although the exact biological mechanisms are still unclear, multiple pathways are likely involved including oxidative stress, systemic inflammation, and/or endocrine disruption (Ha 2021). It has been recently proposed that exposure to air pollution during periods with high developmental plasticity such as pregnancy and perinatal ages could represent one of the major contributors to the rising prevalence of developmental disabilities around the world (Ha 2021).

Other studies explored the potential relationship between exposure to heavy metals such as lead (Pb), mercury (Hg), and manganese (Mn) and ADHD risk/symptoms (Huang et al. 2016; Lee et al. 2018; Muñoz et al. 2020). With this respect, the most recent meta-analysis of cohort studies (Dalla et al. 2022) indicated that children exposed to higher levels of lead and heavy metal pollution in general, are at greater risk of developing ADHD than those exposed to lower levels. In particular, the potentially detrimental effect of Pb has been extensively investigated once the World Health Organization (WHO) identified Pb as one of ten chemicals of major public health concern that require action by member states in order to protect workers, children, and women of reproductive age (Donzelli et al. 2019). Heavy metals, especially Pb, can indeed easily cross the blood–brain barrier (Goodlad et al. 2013) and affect the development of the central nervous system through the binding to –SH residues and displacement of iron, which alters the tertiary structure of enzymes and other proteins. Previous literature has indicated that early exposure to heavy metals could result in changes in fetal programming with either consequences in the pre/perinatal period or triggering alterations in gene expression which may appear later in development (Dietert et al. 2011; Gorini et al. 2014).

Previous research has also explored the possible link between persistent organic pollutants (POPs) and ADHD, in particular measuring the exposure to POPs (e.g., perfluoroalkyl substances PFASs) during pregnancy and ADHD in offspring (Qu et al. 2021). Indeed, the developing fetal central nervous system is the most vulnerable to POP exposure (Qing Li et al. 2006). POPs (e.g., PFAS; perfluorooctane sulfonate, PFOS; or perfluorooctanoic acid, PFOA) include hundreds of organic chemical compounds with common properties, such as long-term persistence, widespread diffusion in the environment, and bioaccumulation in fat tissues of living organisms (Lee et al. 2007). Most hazardous POPs have been banned from the wealthiest countries since 2000 (Lee et al. 2007); some poorer countries still use them such that POP residues are commonly found in animal food, in fat-rich human food and in industrial products (Lee et al. 2007). Evidence from previous systematic reviews is not very thorough (Berghuis et al. 2015; Polańska et al. 2012), with some studies supporting the association between different types of POPs and ADHD (e.g., Hoffman et al. 2010), while some others do not (e.g., Fei et al. 2008).

The association of ADHD with phthalates, which are the most used plasticizers in the world (Petersen and Jensen 2016; Praveena et al. 2020) has also been studied; association has been deemed as possible although the specific impact in ADHD etiology of phthalates needs further analysis (Praveena et al. 2020; Polańska et al. 2012). Due to their ubiquitous presence in the environment, humans can be exposed to phthalates through various pathways (ingestion, inhalation, injection, and absorption through the skin). Phthalates have been reported crossing the placental barrier, even in early pregnancy (Lucaccioni et al. 2021). At this age, the fetal liver’s detoxification system is still unable to convert these metabolites, which may be free to act as endocrine-disrupting chemical signals on fetal development (Lucaccioni et al. 2021).

As outlined above, the currently available evidence is suggestive of association yet inconclusive, as the findings in this field are limited by several methodological reasons, such as the cross-sectional nature of the reviewed studies, their limited sample sizes, and other confounding factors as socioeconomic status (Aghaei et al. 2019; Donzelli et al. 2019; Praveena et al. 2020). It can also be difficult to determine the actual impact of pollutants given the conditions of different countries with the relative exposure to clean air and water, and safe living environments.

The main goal of this overview of reviews was to assess the state of the art on the correlations between environmental pollutant exposure and either ADHD diagnosis or symptoms by collecting and evaluating available systematic reviews and meta-analyses. Therefore, our first aim was to evaluate the state of the art on this topic, with a specific focus on the correlations between environmental pollutants — founded through medical subject heading (MeSH) terms and ADHD. Second, we were also interested in exploring the methodological quality of earlier studies by overviewing systematic reviews and meta-analyses. Although correlational studies does not indicate causality, the present approach (Smith et al. 2011) has been proved to be useful in highlighting associations between factors for future directions of research, also in the field of neurodevelopmental conditions (e.g., Micai et al. 2020).

## Methods

### Search strategy

The protocol for this systematic overview of reviews was registered with PROSPERO: CRD42022341496. Preferred Reporting Items for Systematic Reviews and Meta-analyses (PRISMA) guidelines were used to conduct this overview (Page et al. 2021). Five authors (ER, AC, SDF, MF, and LM) searched 4 bibliographic databases: PubMed, Web of Science, Scopus, and Cochrane Library. A preliminary search was performed to identify the most suitable terms to appropriately address our research question, i.e., collect and assess available systematic reviews and meta-analyses focused on the association between pollutant exposure and either ADHD diagnosis or symptoms, evaluated through quantitative assessment of functioning. We, therefore, explored available MeSH terms and identified the most comprehensive ones to best reflect the topic amplitude and maximize results. After discussion, the authors reached a consensus on final search terms. The definitive search was first launched on November 15, 2021 and updated on December 21, 2021 and used the following terms: pollutants, metals, pesticides, and hydrocarbons, joined by the Boolean operator “OR”, and “attention deficit disorder with hyperactivity”, and “humans”, joined by “AND”. Search strategy details are reported in Table 1. Different search strings were applied in order to mirror the specific functions of each database screened (see Table 1S in Supplementary Material). In PubMed, Web of Science, and Scopus, the filter to limit the search to “Reviews/Systematic reviews” was applied. The search was limited to studies written in English, and no temporal restrictions were applied. Population, intervention, comparison, outcome, and study design (PICOS) domains were used as a search strategy, and articles were included if the following eligibility criteria were matched: (1) population: humans (from perinatal period to adulthood); (2) intervention: exposure to environmental pollutants, pesticides, and metals; (3) comparison: not applicable; (4) outcome: ADHD diagnosis or ADHD symptoms; and (5) study design: systematic reviews or meta-analyses, including at least 25% of studies whose outcomes were ADHD or ADHD symptoms in relation to the exposure to pollutants. We did not focus on the method by which the exposure was assessed in studies (blood or urine, etc.). Articles were excluded on the basis of the following criteria: (1) population: studies that included non-human samples; (2) intervention: studies that focused specifically on the exposure of teratogens and studies that considered metals as nutrients; (3) outcome: studies specifically focused on genetics and studies that did not consider ADHD or ADHD symptoms as main outcome; and (4) study design: articles that were not systematic reviews or meta-analyses and reviews and meta-analyses with less than 25% of articles related to ADHD or ADHD symptoms.

**Table 1:** Summary of the studies

Author (year)	N. of papers (years)	Population range (age range)	Study design	Pollutants assessed	Assessment of ADHD	Assessment of pollutants	Findings
Aghaei et al. (2019)	28 (2009–2018)	174–46940 (4–17)	19 cohort; 7 cross-sectional; 1 one-year follow up	*Air pollution:* NO_2_, SO_2_, Benzene *Particulate matters:* PM10, PM2.5, PM7, PAH, BC/EC *POPs:* PCDD/Fs	Conner’s (CPRS, CPRS-R; K-CPT, CPT-II); Manuals (DSM, Teacher report DSM-IV); Neuropsychological assessment (ANT, WRAML2, KITAP; BASC-2; NES: SAT and SRTT; LDT); SDQ; CBCL; ADHD rating scales (FBB-ADHS); A-TAC.	Blood; urine; air sampling; DWTD; milk samples	Nineteen associations (61.2%) between particulate air pollutants and increased risk of ADHD. 11 associations (54.55%) between gaseous air pollutants and ADHD and 1 association between POPs and ADHD
Donzelli et al. (2019)	17 (2006-2017)	117–2195 (1.6–20)	2 cross-sectional; 5 birth cohort;10 case-control	*Heavy metals:* Pb	Manual (DSM diagnosis).	Blood; urine; analysis of teeth	70.59% of studies reported a positive association between Lead and ADHD.
Forns et al. (2020)	9 (2003–2015)	185-989 Mother–infant pairs (4–11)	9 cohort	*POPs:* PFAS (PFOS, PFOA)	SDQ; CBCL; Registres (NPR; DNHR; DPCR); Manual (DSM-IV diagnosis).	Maternal serum and plasm; breast milk concentrations	No association between PFOS or PFOA and ADHD [AORs ranging from 0.96 (95% *CI*: 0.87, 1.06) to 1.02 (95% *CI*: 0.93, 1.11)]. *OR*s in girls were above 1 and in boys below 1, indicating a possible effect of sex.
Goodlad et al. (2013)	33 (1972–2010)	37–2588 (1–14)	N/A	*Heavy metals:* Pb	Teacher and parent reports	Teeth; blood, urine; X-ray; hair	Small to medium effect sizes of association between inattention and lead (r = .16). Small effect size of association between hyperactivity/impulsivity and lead (*r* = .13). Medium effect size of association between ADHD and lead (Cohen’s *d* = .51)
He et al. (2019)	15 (2006–2016)	43–4704 (N/A)	7 case control; 4 cohort; 4 cross sectional	*Heavy metals:* Pb	Manuals (DSM-IV-R); semi-structured clinical interview, ADHD rating scale (K-ARS; ADHD-RS); Conners (CPT; CPRS); parent and teacher report of ADHD; Hyperactivity Questionnaire.	Blood (Pb levels < 3 μg/dL)	For cohort studies RD was 0.22 (95% *CI*, 0.02, 0.42, *p* < 0.001). For cross-sectional studies *OR* was 0.35 (95% *CI*, 0.26, 0.49, *p* < 0.001). For case-control studies OR was 1.47 (95% *CI*, 1.06, 2.05, *p* < 0.001).
Kalantary et al. (2020)	6 (2011–2018)	242–1257 (5–15)	5 prospective cohort; 1 cross-sectional	*Air pollution:* PAHs	CBCL; Conners (CPRS; CAARS); Manuals (DSM); parental report of ADHD.	Adduct; air sampling; urine	Significant and positive association between PAH and ADHD [(*AOR*) of 2.57(95% *CI* (1.75-3.78)].
Lam et al. (2017)	9 (2009–2015)	62–622 mother infant pairs + 43 children (N/A)	8 prospective birth cohorts; 1 cross-sectional	*POPs:* PBDEs	Manuals (DSM-IV); CBCL; ITSEA; Conners (K-CPT; CPT-II, CADS); behavioral assessment (BASC-II); SDQ.	Blood; breastmilk; serum	9 weak associations (100%) between PBDE and ADHD or ADHD attention-related.
Nilsen and Tulve (2020)	Forse 34, ma forse 28 (2007–2018)	N/A (2-18)	Cohort studies with mixed designs	*Heavy metals*: Pb, Hg, Mn, As *PhPl* *POPs*	Manuals (DSM-IV; DSM-5)	Blood; hair; urine	Significant associations between ADHD specific diagnosis and Pb [*OR* ranged from 2.89 to 5.23 (2.89–5.23, *p* < 0.001)]. The *OR* for Hg exposure and all ADHD outcomes was 2.68 (2.16–3.19, *p* < 0.0001). The *OR* for Mn exposure and all ADHD outcomes was 2.63 (1.27–4.00, *p* < 0.002) For arsenic only one study (100%) reported an association with ADHD. No effect of POPS on ADHD [*OR* ratio was 0.99 (0.96–1.02)].
Polanska et al. (2012)	8 (2008–2011)	215–1400 mother-infant pairs + 188 - 571 children (N/A)	6 cohort; 2 cross-sectional	*PhPl:* BPA *POPs:* PFCs *Air pollution:* PAHs	Behavioral assessment (BNBAS, BASC-2; BASC-PRS); BRIEF; Teacher-rated ADHD-RS; computerized measurements of inattention and impulsivity; mothers’ report of motor and mental development, parental report of diagnosis; CBCL.	Urine; blood; serum; adducts	1 positive association (100%) between particulate air pollution and ADHD. 1 positive association (50%) between POPs and ADHD. 5 positive associations (100%) were found between phthalates and ADHD.
Praveena et al. (2020)	16 (2009–2020)	122–1318 (0–12)	5 cross-sectional; 9 cohort; 2 case control	*PhPl:* DBP, DEP, DMP, BBP, DCHP, DiNP, DOP, DEHP	SDQ; ADHD rating scale (ADHD-RS; Teacher-rated ADHD-RS; SNAP); Behavioral assessment (DBDRS; BASC-2 PRS; CBSQ); CBCL; Manuals (DSM-IV; ICD-10); Temperament (CTTS-R, CMCTQ), BOT-2; BRIEF	Urine; blood	15 positive associations (93.75%) between phthalates and ADHD.
Qu et al. (2021)	9 (2008–2019)	206–10546 (N/A)	5 cohort; 2 cross-sectional; 2 case-control	*POPs:* PFAS (PFOA, PFOS, PFHxS, PFNA, PFDA)	Prevalence rate of ADHD in children	Blood; breastmilk	No associations between PFOA (*OR* = 1.00, 95% *CI* = 0.75–1.25), PFOS (*OR* = 1.01, 95% *C*I = 0.88–1.14), PFHxS (*OR* = 1.08, 95% *CI* = 0.80–1.36), PFNA (*OR* = 1.13, 95% *CI* = 0.99–1.28), PFDA (*OR* = 1.23, 95% *CI* = 0.15–2.32) and ADHD.
Rivollier et al. (2019)	13 (2003–2016	32–2183 (3-15)	N/A	*POPs*: PBDE, HCB, PCB, DDE, PCE, organic solvents; *Air pollution:* NO2; *PhPl:* BPA; *Heavy metal:* Pb	Manual (DSM-IV); Conners (CPRS; CPT); SDQ; ADHD clinical diagnosis; behavioral assessment (BASC-2; BRS BSID-II); CBCL.	Blood; serum; urine; estimation in water; interrogation	1 positive association (100%) was found between particulate air pollution or gaseous air pollution and ADHD. 7 positive associations (87.5%) between POPs and ADHD. 2 positive associations (66.66%) between phthalates and ADHD.
Roth et al. (2014)	9 (2009–2013)	62–2626 (2.5–18)	9 cohort with mixed designs	*POPs:* PBDE, PFC	Maternal and teacher reports	Blood; breastmilk	5 positive associations (55.55%) between POPs and ADHD.
Yoshimasu et al. (2014)	9 (2003–2013)	129-140887 (0–12)	4 case-control; 4 cohort; 1 cross-sectional	*Heavy metals:* Hg	Medical record review; manuals (DSM-IV; ICD-8; ICD-9); SDQ; CBC; Conners (CPRS)	Blood, industrial release to environments in 1998; maternal fish consumption; hair; VAERS	Summary AOR (95% CI) for the association between methylmercury and ADHD was 1.60 (1.10–2.33).

See supplementary materials for abbreviations of pollutants and assessments

*AOR* adjusted odds ratio, *CI* confidence intervals, *OR* odds ratio, *RD* risk difference

### Selection process

All record titles and abstracts retrieved from the database search were screened by four blinded reviewers (ER, SDF, MF, and LM) who excluded studies that did not meet the eligibility criteria (see supplementary materials for a list of excluded articles). The same authors proceeded with the full-text screening of retained papers according to the inclusion criteria. In case of discordant opinions, the three authors voted to reach a decision. Based on AMSTAR 2 recommendations (Shea et al. 2017), the group of authors included experts in the field, directly involved in each phase of the study.

### Data extraction

After a preliminary work of inter-rater calibration, three reviewers (SDF, MF, and LM) extracted data from the full texts independently. For each systematic review or meta-analysis, we extracted data on: the year of publication, size, and type of population, study design, pollutants considered and means of exposure, modality of ADHD assessment, and main findings on the association between pollutant exposure and ADHD (diagnosis or ADHD-related symptomatology). Table 1 (see “Results”) summarizes all the extracted data. Three authors (SDF, MF, and LM) resolved discrepancies through discussion in order to achieve unanimity.

### Quality assessment of the evidence

The AMSTAR 2 (A MeaSurement Tool to Assess Systematic Reviews) checklists (Shea et al. 2017) were used by three authors (SDF, MF, and LM) to assess the quality of each work that was eligible for the present overview. Discrepancies were resolved after a discussion in order to achieve unanimity. The AMSTAR 2 checklist is a 16-item tool developed to lead an evaluation of methodological quality of systematic reviews, including randomized and non-randomized trials of healthcare interventions. The checklist included questions on: inclusion of PICOS components, presence of a protocol registered before the beginning, justification of the study design’s inclusion, relevance of the literature search, number of authors (at least two needed for inclusion), justification for excluded studies, detailed description of included studies, evaluation of bias risk of the studies included, account of funding for included studies, suitability of methods used for meta-analysis, evaluation of the bias risk in individual studies on the reported results, consideration of the risk of interpreting the results, evaluation of publication bias, and report of funding or conflict of interest. For each question, authors answered “yes,” if the requirements indicated were satisfied; “no,” if requirements were not satisfied, and “partial yes,” if only a few requirements were met.

### Overlap

One of the main issues in performing an overview of reviews is taking into account inter-review overlaps when interpreting results. To this end, we conducted a systematic evaluation of the degree of overlap using the Corrected Covered Area (CCA) approach (Pieper et al. 2014). The interpretation of the CCA was conducted following the overlap categories explained in Pieper et al. (2014) (details in Box 1).

**Box 1** Corrected Covered Area (CCA).

**Table:** 

$$CCA=\frac{N-r}{r\ c-r}$$	
*N =* total number of included publications (including double counting)	
*r =* number of index publications (rows in matrix)	
*c =* number of reviews (column in matrix)	
Interpretation of CCA (%) - 0–5: Slight - 6–10: Moderate - 11–15: High - > 15: Very high	

## Results

### Description of studies

Our search strategy returned 1802 studies (PubMed: 1474 results; Web of Science: 94; Scopus: 234), of which 1644 were excluded using automated filters. Searching on Cochrane Library did not produce any results. For PubMed we used the filter for meta-analyses and systematic reviews, whereas in Web of Science and Scopus we used the filter for reviews. Twenty-five duplicates have been further excluded manually, leading to a total of 133 studies screened.

**Fig. 1:**
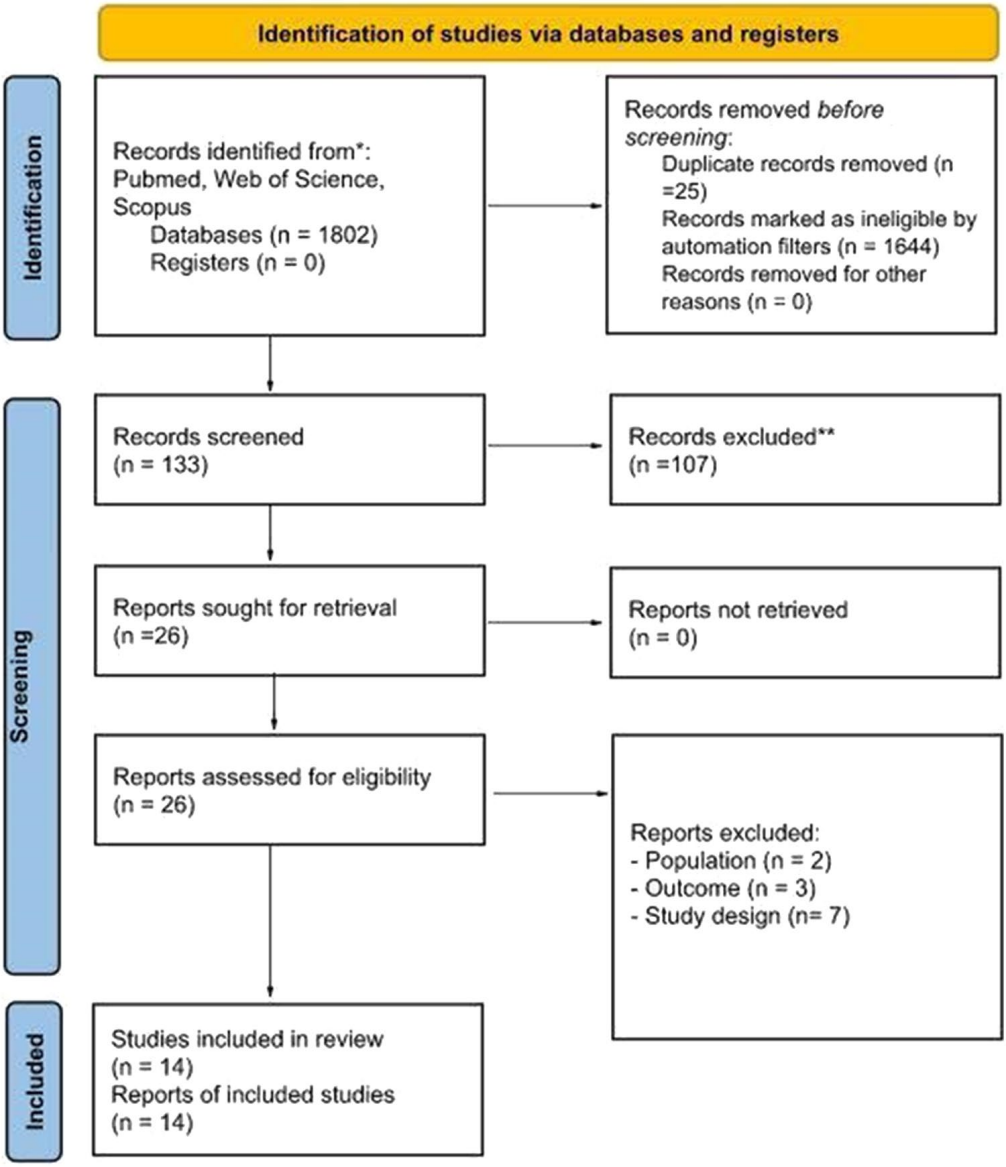
PRISMA flow diagram. *Consider, if feasible to do so, reporting the number of records identified from each database or register searched (rather than the total number across all databases/registers).  **If automation tools were used, indicate how many records were excluded by a human and how many were excluded by automation tools

Four authors (ER, SDF, MF, and LM) screened titles and abstracts, excluding an additional 107 studies. The remaining 26 studies were then screened in their full-text by three authors (SDF, MF, and LM). Studies were excluded at this stage, if they included animal samples (*n* = 2), did not focus on ADHD as an outcome measure (*n* = 3), and were not a systematic review or included less than 25% of articles focused specifically on ADHD (*n*= 7). After the selection, 14 studies were retained in the present overview: 6 systematic reviews, 3 meta-analyses, and 5 systematic reviews and meta-analyses. Process: Figure 1 provides the process of records’ identification and screening and the eligibility and inclusion actions, and Table 1 summarizes all the extracted data.

### Assessment of quality

**Fig. 2:**
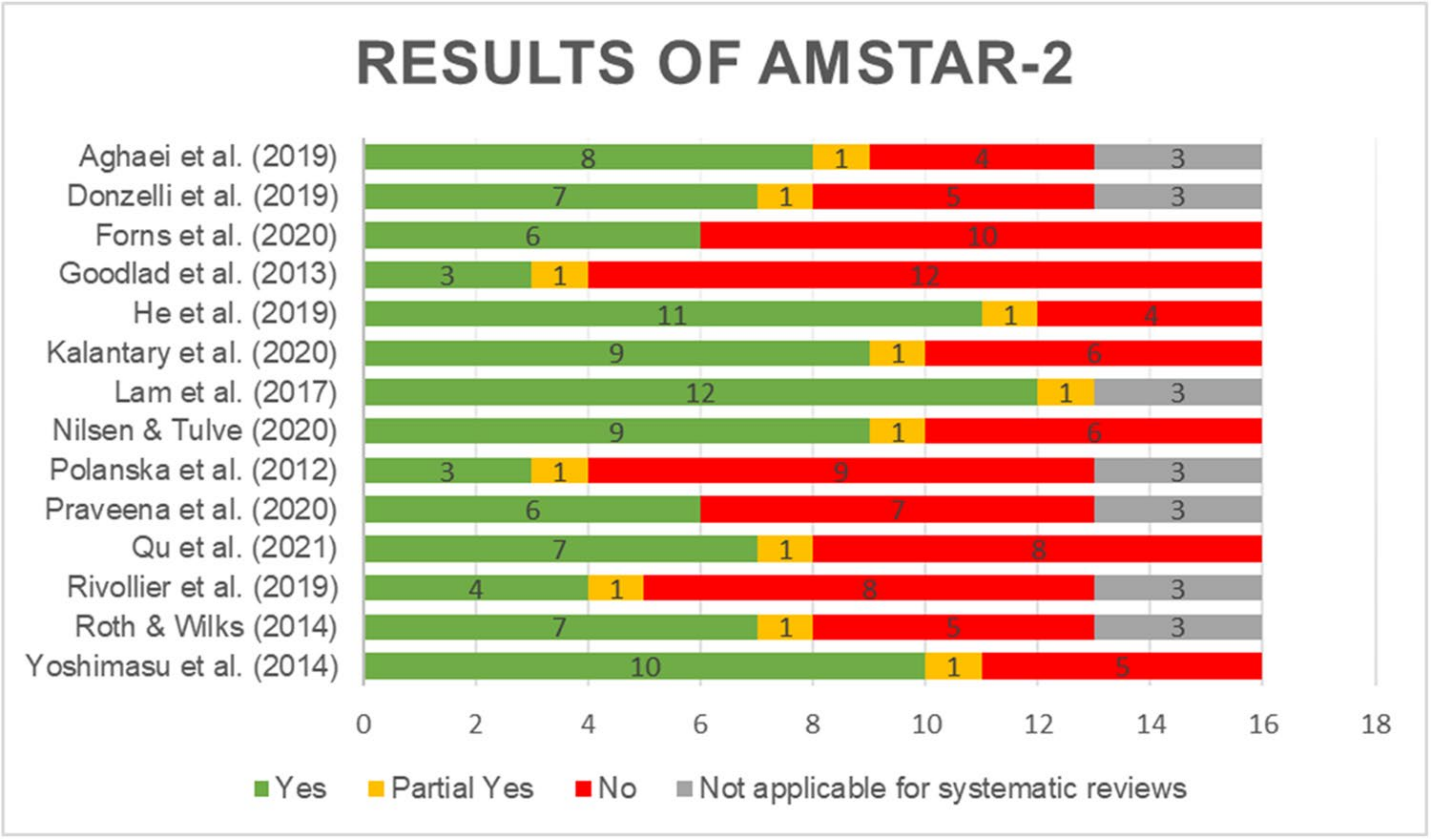
Assessment of quality results

The overall risk of bias ratings for systematic reviews ranged from 3.5 to 12.5 (*mean* = 7.14; *standard deviation* = 2.95) and for meta-analyses ranged from 3.5 to 1.5 (*mean* = 8.29; *standard deviation* = 2.80). Except for one study, which has high methodological quality, the overall quality was rated as critically low. According to Shea et al.’s (2017) classification, the items that most affect the quality of studies were the absence of a written protocol or guide to follow before the start of the review (item 2), the lack of a comprehensive literature search strategy (item 4), the lack of a list of excluded articles and a justification for their exclusion (item 7), the absence of a technique for assessing the risk of bias in individual studies (item 9), inappropriate methods for the statistical combination of results (item 11), no consideration of the risk of bias when interpreting the results of the review (item 13), and a lack of an evaluation of publication bias (item 15). Most of the included studies presented weaknesses in several crucial items, with particular respect to items 2 and 7. Only one study indeed reported the presence of a written protocol and a list of excluded studies (Lam et al. 2017). AMSTAR-2 results are summarized in Fig. 2 (AMSTAR-2 full scores are available in supplementary materials, Table 7S).

### Overlap

We conducted a systematic evaluation of the degree of overlap of the studies. Even though all the studies included in Kalantary et al.’s (2020) review overlapped with those from other reviews and meta-analyses, the CCA value of the present overview was 2.3%, indicating a modest degree of overlap.

### Participants

The included articles have considered studies with different designs, including birth cohort, cohort, cross-sectional, and case-control studies.

With regard to the population, one work included articles involving mother-infant pairs (Forns et al. 2020), 10 works included articles that recruited only children (Aghaei et al. 2019; Donzelli et al. 2019; Goodlad et al. 2013; He et al. 2019; Kalantary et al. 2020; Nilsen and Tulve 2020; Qu et al. 2021; Rivollier et al. 2019; Roth and Wilks 2014; Yoshimasu et al. 2014), and 3 works considered articles involving both children and mother–infant pairs (Lam et al. 2017; Polańska et al. 2012; Praveena et al. 2020). Most of the included studies were conducted either in North America (10 studies) or in Europe (11 studies). Other studies were conducted in Asia (6 studies in East Asia, 1 in South Asia, and 2 in Middle East). None of the studies considered participants from Africa, South America, or Oceania.

### Pollutants

Regarding the frequency of pollutants, POPs are considered in 8 studies (Aghaei et al. 2019; Forns et al. 2020; Lam et al. 2017; Nilsen and Tulve 2020; Polańska et al. 2012; Qu et al. 2021; Rivollier et al. 2019; Roth and Wilks 2014). Heavy metals were considered in 5 studies (Donzelli et al. 2019; Goodlad et al. 2013; He et al. 2019; Nilsen and Tulve 2020; Yoshimasu et al. 2014). Air pollutants were measured in 4 studies (Aghaei et al. 2019; Kalantary et al. 2020; Polanska et al. 2012; Rivollier et al. 2019), and 3 studies considered phthalates (Polańska et al. 2012; Praveena et al. 2020; Rivollier et al. 2019).

### Assessment of pollutants

Different methods were used to assess the pollutants’ exposure. Blood sample was the most used method, as reported in 12 studies (Aghaei et al. 2019; Donzelli et al. 2019; Goodlad et al. 2013; He et al. 2019; Lam et al. 2017; Nilsen and Tulve 2020; Polańska et al. 2012; Praveena et al. 2020; Qu et al. 2021; Rivollier et al. 2019; Roth and Wilks 2014; Yoshimasu et al. 2014), followed by urine samples in 8 studies (Aghaei et al. 2019; Donzelli et al. 2019; Goodlad et al. 2013; Kalantary et al. 2020; Nilsen and Tulve 2020; Polańska et al. 2012; Praveena et al. 2020; Rivollier et al. 2019). Five studies referred to breast milk concentrations (Aghaei et al. 2019; Forns et al. 2020; Lam et al. 2017; Qu et al. 2021; Roth and Wilks 2014); 4 studies to serum (Forns et al. 2020; Lam et al. 2017; Polanska et al. 2012; Rivollier et al. 2019); and 2 studies to ambient or air sampling (Aghaei et al. 2019; Kalantary et al. 2020), adducts (Kalantary et al. 2020; Polańska et al. 2012), and plasma (Aghaei et al. 2019; Forns et al. 2020). Finally, 4 studies reported other biological proxies, such as X-ray, hair, and teeth exposure (Donzelli et al. 2019; Goodlad et al. 2013; Nilsen and Tulve 2020; Yoshimasu et al. 2014), and 3 studies used other procedures like estimation in water, interrogation, or industrial release to environments (Aghaei et al. 2019; Rivollier et al. 2019; Yoshimasu et al. 2014).

### Assessment of ADHD

Except for three, all studies considered a clinical ADHD diagnosis based either on Diagnostic and Statistical Manual of Mental Disorders (DSM) or International Classification of Diseases (ICD) criteria (Aghaei et al. 2019; Donzelli et al. 2019; Forns et al. 2020; He et al. 2019; Kalantary et al. 2020; Lam et al. 2017; Nilsen and Tulve 2020; Polańska et al. 2012; Praveena et al. 2020; Rivollier et al. 2019; Yoshimasu et al. 2014).

Most studies used questionnaires to evaluate ADHD symptoms: 11 studies used parent-report (Aghaei et al. 2019; Forns et al. 2020; Goodlad et al. 2013; He et al. 2019; Kalantary et al. 2020; Lam et al. 2017; Polańska et al. 2012; Praveena et al. 2020; Rivollier et al. 2019; Roth and Wilks 2014; Yoshimasu et al. 2014), 6 teacher-report (Aghaei et al. 2019; Goodlad et al. 2013; Lam et al. 2017; Polańska et al. 2012; Praveena et al. 2020; Roth and Wilks 2014) and 1 self-report (Kalantary et al. 2020). Five studies also used a neuropsychological test to assess the cognitive domain of ADHD (Aghaei et al. 2019; Lam et al. 2017; Polańska et al. 2012; Praveena et al. 2020; Rivollier et al. 2019). Other assessment measures were interviews (He et al. 2019), psychological test (Lam et al. 2017), observational tools (Polańska et al. 2012; Rivollier et al. 2019), or population-based registries (Forns et al. 2020). Only one study considered the prevalence rate of ADHD (Qu et al. 2021).

### Exposure to pollutants and ADHD

#### Air pollution

##### Particulate air pollution

Three systematic reviews considered the association between air pollution and ADHD (Aghaei et al. 2019; Polańska et al. 2012; Rivollier et al. 2019). A positive association was observed in 63.64% of cases (21/33). Aghaei et al. (2019) reported 19 positive associations in 31 investigations, whereas the other two studies considered only 1 investigation each. Only 1 meta-analysis (Kalantary et al. 2020) was performed to evaluate the association between polycyclic aromatic hydrocarbons (PAH) and either symptoms or diagnosis of ADHD, showing a significant and positive association, with an adjusted odds ratio (*AOR*) of 2.57 (95% *CI* = 1.75–3.78).

We included in this section also PAH, despite being a large group of organic compounds that can be classified in different subgroups. However, in the systematic reviews/meta-analyses examined here, PAHs were considered as an air pollutant or particulate air pollutant.

##### Gaseous air pollution

Two systematic reviews considered the association between gaseous air pollution and ADHD (Aghaei et al. 2019; Rivollier et al. 2019). Overall, in 56.52% of cases (12/23), a positive association was reported. Most of the associations were included in the study of Aghaei et al. (2019), reporting 11 associations in 22 investigations.

Aghaei’s review also found an association between PCDD, which was considered an air pollutant by the authors and ADHD symptoms (Aghaei et al. 2019). Based on the previous literature, we consider PCDD a persistent organic pollutant in the present work.

#### Heavy metal

##### Lead

Only one systematic review considered the association between Pb and ADHD, showing 12 positive associations in 17 investigations (70.59%; Donzelli et al. 2019). Most of the included studies on Pb were meta-analyses. Goodlad et al. (2013) considered the association between the inattention symptom and Pb, reporting a positive correlation of 0.16 (95% *CI* = 0.12–0.20, *Z* = 8.09, *p* <.001) across 27 studies. The correlation between hyperactivity/impulsivity and Pb was 0.13 across 23 studies (95% *CI* = 0.09–0.16, *Z* = 7.22, *p* <.001). For associations between ADHD and Pb, Cohen’s *d* across 9 studies was 0.51 (95% *CI* = 0.35, *Z* = 6.33, *p* < .001). He et al. (2019) considered 4 cohort studies, showing a risk difference (*RD*) of 0.22 (95% *CI* = 0.02–0.42, *p* < .001). Furthermore, they reported an odds ratio (*OR*) of 0.35 (95% *CI* = 0.26–0.49, *p* < .001) across 4 cross-sectional studies and an *OR* of 1.47 (95% *CI* = 1.06–2.05, *p* < .001) across 7 case-control studies. Nilsen and Tulve (2020) considered the association between all subtypes of ADHD and Pb, reporting an *OR* of 3.39 for 12 studies (90% *CI* = 2.66–4.12, *p* < .001); when considering specific ADHD subtypes and Pb, the *OR* varied from 2.89 to 5.23 (*CI* = 2.89–5.23, *p* <.001).

##### Manganese and mercury

Two meta-analyses examined the association between Mn and Hg and ADHD. Nilsen and Tulve’s (2020) meta-analysis included 3 studies, depicting an *OR* of 2.68 (*CI* = 2.16–3.19, *p* < .0001) for Hg exposure and all ADHD outcomes. Moreover, the authors reported an *OR* of 2.63 (*CI* = 1.27–4.00, *p* < .002) for Mn exposure and all ADHD outcomes. Yoshimasu et al. (2014) conducted a meta-analysis on 2 studies related to methylmercury, reporting an *OR* of 1.60 (95% *CI* = 1.10–2.33, *p* value not reported).

##### Other heavy metals

Only one systematic review considered the association between arsenic and ADHD reporting a positive association in 1 investigation (Nilsen and Tulve 2020).

#### Persistent organic pollutants

Five systematic reviews investigated the association between POPs and ADHD. Overall, 22 positive associations out of 28 investigations (78.57%) were observed. Lam et al. (2017) showed 9 weak associations for 9 investigations; Roth and Wilks (2014) 5 positive associations for 9 investigations; Rivollier et al. (2019) 7 positive associations for 8 investigations; and Polańska et al. (2012) 1 positive association for 2 investigations; and Aghaei et al. (2019) found 1 positive association for 2 investigations. Three meta-analyses investigated the association between POPs and ADHD (Forns et al. 2020; Nilsen and Tulve, 2020; Qu et al. 2021), none of them finding significant associations. However, Forns et al. (2020) found a possible effect of sex with *OR*s in females above 1 (ranging from 1.12 (95% *CI* = 0.87–1.06) to 1.30 (95% *CI* = 0.98–1.73)), and below 1 in males (ranging from 0.92 (95% *CI* = 0.81–1.03) to 1.03 (95% *CI* = 0.85–1.25)). Moreover, Qu et al. (2021) observed possible regional differences in the association between PFOS and ADHD, showing a positive correlation limited to the USA (*OR* = 1.05, 95% *CI* = 1.02–1.08).

#### Phthalates

Three systematic reviews considered the association between phthalates and ADHD (Praveena et al. 2020; Polańska et al. 2012; Rivollier et al. 2019). Overall, 22 positive associations in 24 investigations (91.96%) were reported between phthalates and ADHD. Specifically, Praveena et al. (2020) found 15 positive associations out of 16 investigations, whereas Polańska et al. (2012) and Rivollier et al. (2019) showed positive associations on all the investigations considered (5/5 and 2/2, respectively). The meta-analysis of Nilsen and Tulve (2020) found an *OR* of 3.31 (95% *CI* = 2.59–4.02, *p* < .0001) for the association between phthalates and ADHD. However, the considerable heterogeneity of the included studies needs to be considered when interpreting the results.

## Discussion

The current overview investigated existing systematic reviews and meta-analyses focused on the potential association between exposure to environmental pollutants and either ADHD diagnosis or symptoms. To this end, more than 1800 studies were screened. The eligible studies were 14, of which 3 were meta-analyses, 4 systematic reviews and meta-analyses, and 7 systematic reviews.

We found several pollutants through medical subject heading (MeSH) terms and, for the sake of critical investigation and discussion, we categorized them in 4 groups with relative sub-groups: air pollution (both particulate and gaseous air pollution); heavy metals (Pb, Mn, Hg, and other heavy metals); persistent organic pollutants; and phthalates. Some articles concurrently focused on multiple pollutants, whereas some others analyzed only one kind of pollutant.

With respect to the first group, air pollution, we found a positive association between air pollution and increased risk of ADHD/symptoms of ADHD in about 60% of the total investigations included (Aghaei et al. 2019; Polańska et al. 2012; Rivollier et al. 2019). The only meta-analysis found a significant positive association between PAHs and ADHD (Kalantary et al. 2020).

This said, most of the studies included in these three reviews and in the meta-analysis exclusively considered ADHD symptoms and attention problems, using either self-report questionnaires or neuropsychological tests (Kalantary et al. 2020; Polańska et al. 2012; Rivollier et al. 2019). Only in Kalantary et al.’s (Kalantary et al. 2020) and Aghaei et al.’s (Aghaei et al. 2019) studies, there was only a limited set of studies that considered ADHD diagnosis with clinical confirmation, most of the associations being with symptoms rather than with diagnostic ADHD status.

Our findings about POPs are controversial. Whereas 4 systematic reviews (Aghaei et al. 2019; Polańska et al. 2012; Rivollier et al. 2019; Roth and Wilks 2014) reported a positive association between these pollutants and ADHD, the other 4 reviews and none of the meta-analyses found a significant association (Forns et al. 2020; Lam et al. 2017; Nilsen and Tulve 2020; Qu et al. 2021). Furthermore, the reviews underlining the correlation between POPs and ADHD included almost all studies that explored symptoms of ADHD and not diagnostic outcomes of it. A possible explanation for those divergent results is the remarkable heterogeneity within the POP category, with several compounds independently assessed across studies.

With regard to heavy metals and phthalates, our overview revealed concurrent positive associations with both ADHD status and symptoms. This finding differed from results regarding other pollutants for the notable convergence across studies (Donzelli et al. 2019; Goodlad et al. 2013; He et al. 2019; Nilsen and Tulve 2020; Polanska et al. 2012; Praveena et al. 2020; Yoshimasu et al. 2014). Moreover, most reviews or meta-analyses on these pollutants included studies evaluating ADHD through caregiver reports, whereas most of the studies included in the 3 meta-analyses (of which 2 focused on Pb, Goodlad et al. 2013, He et al. 2019, and 1 on multiple heavy metals and phthalates, Nilsen and Tulve 2020) considered clinical diagnoses of ADHD. With due precautions, the findings of this overview indicate these categories of environmental pollutants are the most associated with ADHD diagnostic status. Our findings are also in line with those of the very recent meta-analysis of Dalla et al. (2022), which suggests a significant relationship between the risk of developing ADHD and exposure to lead and, more in general, with heavy metal pollution. Nonetheless, it is important to note that the present work differs in some relevant methodological aspects from one of Dalla and colleagues. First, this study is an overview of reviews and meta-analyses, whereas Dalla and others performed a meta-analysis of cohort studies. Furthermore, we employed a different research string from the one used by Dalla and colleagues. Those differences led to an only marginal overlap between the present and Dalla et al.’s studies (only 9 out of 21 studies analyzed by Dalla and colleagues are included within our overview).

Alongside the analysis of specific environmental pollutants, the present overview offers insights about the methodological quality of the inspected reviews and meta-analyses. For this purpose, we used the AMSTAR 2 checklists (Shea et al. 2017). The overall ratings of risk bias were generally judged as critically low, except for 1 study (Lam et al. 2017), indicating unsatisfactory methodological quality in the literature. Therefore, the majority of the studies included in this work presented weaknesses in AMSTAR 2 crucial items. In particular, eligible studies lacked both protocol registrations before starting the literature search and written protocols/lists of the excluded studies. Beyond the weaknesses highlighted by AMSTAR 2 checklists, each of the reviews and meta-analyses we considered exhaustively acknowledged the methodological limitations and possible biases of their results, recommending caution and non-causal associations between environmental pollutants and ADHD, as well as suggesting more in-depth studies. Given these considerations, at the current stage there is too limited evidence to determine causality for the relationships between environmental pollutants and ADHD.

Starting from the observed limitations, future meta-analyses and systematic reviews in this field should carefully consider the use of critical appraisal tools, such as AMSTAR 2 checklist, to significantly improve the methodological quality of the metanalytical work, and especially register their study to PROSPERO. Although the study of environmental variables is extraordinarily complex in cause-and-effect analysis, greater rigor in the application of guidelines makes it more possible to draw conclusions. In addition, future studies should focus on a narrower range of pollutants, such as heavy metals or phthalates, to increase the chance of highlighting more solid results. Finally, even within the same category of pollutants, consistency in the design — especially for longitudinal studies — and in the study characteristics, such as timing of exposure, outcome measurement, and ADHD assessment, is needed to reach definitive conclusions. In particular, the homogeneity of the exposure time and the considered levels of pollutants are important methodological elements to compare results from different studies. Given that the most critical windows for the detrimental effects on the brain are pregnancy and the perinatal period, in-depth studies on exposure to environmental pollutants within this time frame are recommended.

It is also crucial to mention that the reviews and meta-analyses considered here did not include investigations from Oceania, South America, and Africa. This is a limit that needs to be solved to assess potential differences or similarities between countries in terms of degree of exposure to environmental pollutants. Indeed, socio-economics factors play a central role in determining the level of environmental pollutants and their potential impact on neurodevelopment at early stages.

Lastly, most of the studies on heavy metals did not wholly consider the fact that nutritionally essential metals may significantly modify health risks related to exposure to non-essential toxic metals (Goyer 1997). As an example, magnesium supplementation has been demonstrated to have protective effects against cadmium accumulation in the body (Matović et al. 2010). Thus, adequate nutrition may partly compensate for the exposure to some environmental pollutants heavy metals. In light of these considerations, the need to focus on which countries in the study is carried out remains crucial, in addition to the dose, route of exposure, time of exposure of the environmental pollutant, and the nutritional status of the subject.

Our work represents the first systematic overview in the field of the exposure to environmental pollutants and ADHD. We underscored some preliminary results and considered the quality of the studies discussing weaknesses and offering suggestions for future research. Our overview also presents some limitations. Firstly, we used the filter for meta-analyses and systematic reviews in our search strategy, resulting in reductions in the articles considered. While being widely used in overview works, this criterion could have limited the identification of eligible studies. Furthermore, as we were interested in exploring the possible role of pollutants with specific respect to the clinical phenotype of ADHD, studies more focused on broader manifestation of the condition were not included in this study.

Secondly, we did not consider the period of exposure to pollutants, given that this information was seldom reported in the reviews/meta-analyses considered. Nevertheless, this datum is critical to compare and correctly interpret the results of these studies. Secondly, we did not include correlations or analyses because of the nature of the overview, but umbrella review with meta-analysis can be a future development of the present work.

## Conclusion

This overview of review and meta-analyses suggests a significant role for some pollutants, in particular heavy metals and phthalates, in the increased risk of developing ADHD symptoms. However, at the current stage, data from existing literature also do not allow to weight the role of the different environmental pollutants. In addition, this overview offers some suggestions for conducting reviews and meta-analyses in this specific area.

### Supplementary Information

ESM 1 (DOCX 299 kb)

## Data Availability

Not applicable for that section

## Code availability

Not applicable for that section

## References

[CR1] Aghaei M, Janjani H, Yousefian F, Jamal A, Yunesian M (2019). Association between ambient gaseous and particulate air pollutants and attention deficit hyperactivity disorder (ADHD) in children: a systematic review. Environ Res.

[CR2] American Psychiatric Association (2013). Diagnostic and Statistical Manual for Mental Disorders (5th ed).

[CR3] Berghuis SA, Bos AF, Sauer PJJ (2015). Developmental neurotoxicity of persistent organic pollutants: an update on childhood outcome. Arch Toxicol.

[CR4] Cabral M, Liu S, Soares N (2020) Attention-deficit/hyperactivity disorder: diagnostic criteria, epidemiology, risk factors and evaluation in youth. Transl Pediatr 9:S104–S113. 10.21037/tp.2019.09.08 10.21037/tp.2019.09.08 PMC7082246 32206588

[CR5] Cheslack-Postava K, Rantakokko P, Kiviranta H, Hinkka-Yli-Salomäki S, Surcel HM, Vivio N (2022). Maternal serum persistent organic pollutant exposure and offspring diagnosed ADHD in a national birth cohort. Environ Res.

[CR6] Dalla MDB, Ayala CO, Castro FCDAQ, Neto FK, Zanirati G, Canon-Montanez W, Mattiello R (2022). Environmental pollution and attention deficit hyperactivity disorder: a meta-analysis of cohort studies. Environ Pollut.

[CR7] Data and statistics about ADHD. Centers for Disease and Control Prevention (2019) Available online: https://www.cdc.gov/ncbddd/adhd/data.html

[CR8] Dietert RR, Dietert JM, DeWitt JM (2011). Environmental risk factors for autism. Emerg Health Treats J.

[CR9] Donzelli G, Carducci A, Llopis-Gonzalez A, Verani M, Llopis-Morales A, Cioni L, Morales-Suárez-Varela M (2019). The association between lead and attention-deficit/hyperactivity disorder: a systematic review. Int J Environ Res Public Health.

[CR10] Fei C, McLaughlin JK, Lipworth L, Olsen J (2008). Prenatal exposure to perfluorooctanoate (PFOA) and perfluorooctanesulfonate (PFOS) and maternally reported developmental milestones in infancy. EHP.

[CR11] Forns J, Stigum H, Høyer BB, Sioen I, Sovcikova E, Nowack N (2018). Prenatal and postnatal exposure to persistent organic pollutants and attention-deficit and hyperactivity disorder: a pooled analysis of seven European birth cohort studies. Int J Epidemiol.

[CR12] Forns J, Verner MA, Iszatt N, Nowack N, Bach CC, Vrijheid M (2020). Early life exposure to perfluoroalkyl substances (PFAS) and ADHD: a meta-analysis of nine European population-based studies. Environ Health Perspect.

[CR13] Gorini F, Muratori F, Morales MA (2014). The role of heavy metal pollution in neurobehavioral disorders: a focus on autism. Rev J Autism Dev Disord.

[CR14] Goodlad JK, Marcus DK, Fulton JJ (2013). Lead and attention-deficit/hyperactivity disorder (ADHD) symptoms: a meta-analysis. Clin Psychol Rev.

[CR15] Goyer RA (1997). Toxic and essential metal interactions. Annu Rev Nutr.

[CR16] Ha S (2021). Air pollution and neurological development in children. DMCN.

[CR17] He J, Nin H, Huang R (2019). Low blood lead levels and attention-deficit hyperactivity disorder in children: a systematic review and meta-analysis. Environ Sci Pollut Res Int.

[CR18] Hoffman K, Webster TF, Weisskopf MG, Weinberg J, Vieira VM (2010). Exposure to polyfluoroalkyl chemicals and attention deficit/hyperactivity disorder in US children 12–15 years of age. EHP.

[CR19] Huang S, Hu H, Sánchez BN, Peterson KE, Ettinger AS, Lamadrid-Figueroa H (2016). Childhood blood lead levels and symptoms of attention deficit hyperactivity disorder (ADHD): a cross-sectional study of Mexican children. EHP.

[CR20] Kalantary R, Jaffarzadeh N, Rezapour M, Hesami Arani M (2020). Association between exposure to polycyclic aromatic hydrocarbons and attention deficit hyperactivity disorder in children: a systematic review and meta-analysis. Environ Sci Pollut Res.

[CR21] Kim JH, Kim JY, Lee J, Jeong GH, Lee E, Lee S (2020). Environmental risk factors, protective factors, and peripheral biomarkers for ADHD: an umbrella review. Lancet Psychiatry.

[CR22] Lam J, Lanphear BP, Bellinger D, Axelrad DA, McPartland J, Sutton P (2017). Developmental PBDE exposure and IQ/ADHD in childhood: a systematic review and meta-analysis. Environ Health Perspect.

[CR23] Lee MJ, Chou MC, Chou WJ, Huang CW, Kuo HC, Lee SY, Wang LJ (2018). Heavy metals’ effect on susceptibility to attention-deficit/hyperactivity disorder: implication of lead, cadmium, and antimony. Int J Environ Res Public Health.

[CR24] Lee DH, Jacobs DR, Porta M (2007). Association of serum concentrations of persistent organic pollutants with the prevalence of learning disability and attention deficit disorder. J Epidemiol Community Health.

[CR25] Leopold DR, Christopher ME, Olson RK, Petrill SA, Willcutt EG (2019). Invariance of ADHD symptoms across sex and age: a latent analysis of ADHD and impairment ratings from early childhood into adolescence. J Abnorm Child Psychol.

[CR26] Lucaccioni L, Trevisani V, Passini E, Righi B, Plessi C, Predieri B, Iughetti L (2021). Perinatal exposure to phthalates: from endocrine to neurodevelopmental effects. Int J Mol Sci.

[CR27] Luo Y, Weibman D, Halperin JM, Li X (2019). A review of heterogeneity in attention deficit/hyperactivity disorder (ADHD). Front Hum Neurosci.

[CR28] Matović V, Bulat ZP, Djukić-Ćosić D, Soldatović D (2010). Antagonism between cadmium and magnesium: a possible role of magnesium in therapy of cadmium intoxication. Magnes Res.

[CR29] Micai M, Fulceri F, Caruso A, Guzzetta A, Gila L, Scattoni ML (2020). Early behavioral markers for neurodevelopmental disorders in the first 3 years of life: an overview of systematic reviews. Neurosci Biobehav Rev.

[CR30] Myhre O, Låg M, Villanger GD, Oftedal B, Øvrevik J, Holme JA (2018). Early life exposure to air pollution particulate matter (PM) as risk factor for attention deficit/hyperactivity disorder (ADHD): need for novel strategies for mechanisms and causalities. Toxicol Appl Pharmacol.

[CR31] Muñoz MP, Rubilar P, Valdés M, Muñoz-Quezada MT, Gómez A, Saavedra M, Iglesias V (2020). Attention deficit hyperactivity disorder and its association with heavy metals in children from northern Chile. Int J Hyg Environ Health.

[CR32] Nigg J, Nikolas M, Burt SA (2010). Measured gene-by-environment interaction in relation to attention-deficit/hyperactivity disorder. J Am Acad Child Adolesc Psychiatry.

[CR33] Nilsen FM, Tulve NS (2020). A systematic review and meta-analysis examining the interrelationships between chemical and non-chemical stressors and inherent characteristics in children with ADHD. Environ Res.

[CR34] Page MJ, McKenzie JE, Bossuyt PM (2021). The PRISMA 2020 statement: an updated guideline for reporting systematic reviews. BMJ.

[CR35] Petersen JH, Jensen LK (2016). Phthalates in soft PVC products used in food production equipment and in other food contact materials on the Danish and the Nordic Market 2013-2014. Int J Food Contam.

[CR36] Pieper D, Antoine SL, Mathes T, Neugebauer EA, Eikermann M (2014). Systematic review finds overlapping reviews were not mentioned in every other overview. J Clin Epidemiol.

[CR37] Polańska K, Jurewicz J, Hanke W (2012). Exposure to environmental and lifestyle factors and attention-deficit/hyperactivity disorder in children—a review of epidemiological studies. Int J Occup Med Environ Health.

[CR38] Posner J, Polanczyk GV, Sonuga-Barke E (2020). Attention-deficit hyperactivity disorder. Lancet.

[CR39] Praveena SM, Munisvaradass R, Masiran R, Rajendran RK, Lin CC, Kumar S (2020). Phthalates exposure and attention-deficit/hyperactivity disorder in children: a systematic review of epidemiological literature. Environ Sci Pollut Res Int.

[CR40] Qing Li Q, Loganath A, Seng Chong Y, Tan J, Philip Obbard J (2006). Persistent organic pollutants and adverse health effects in humans. J Toxicol Environ Health.

[CR41] Qu A, Cao T, Li Z, Wang W, Liu R, Wang X (2021). The association between maternal perfluoroalkyl substances exposure and early attention deficit hyperactivity disorder in children: a systematic review and meta-analysis. Environ Sci Pollut Res Int.

[CR42] Rivollier F, Krebs MO, Kebir O (2019). Perinatal exposure to environmental endocrine disruptors in the emergence of neurodevelopmental psychiatric diseases: a systematic review. Int J Environ Res Public Health.

[CR43] Roth N, Wilks MF (2014). Neurodevelopmental and neurobehavioural effects of polybrominated and perfluorinated chemicals: a systematic review of the epidemiological literature using a quality assessment scheme. Toxicol Lett.

[CR44] Sayal K, Prasad V, Daley D, Ford T, Coghill D (2018). ADHD in children and young people: prevalence, care pathways, and service provision. Lancet Psychiatry.

[CR45] Shea BJ, Reeves BC, Wells G, Thuku M, Hamel C, Moran J (2017). AMSTAR 2: a critical appraisal tool for systematic reviews that include randomised or non-randomised studies of healthcare interventions, or both. BMJ.

[CR46] Smith V, Devane D, Begley CM, Clarke M (2011). Methodology in conducting a systematic review of systematic reviews of healthcare interventions. BMC.

[CR47] Thapar A, Cooper M, Eyre O, Langley K (2013). Practitioner review: what have we learnt about the causes of ADHD?. J Child Psychol Psychiatry.

[CR48] Thapar A, Cooper M (2016). Attention deficit hyperactivity disorder. Lancet.

[CR49] Yoshimasu K, Kiyohara C, Takemura S, Nakai K (2014). A meta-analysis of the evidence on the impact of prenatal and early infancy exposures to mercury on autism and attention deficit/hyperactivity disorder in the childhood. Neurotoxicology.

[CR50] Zgodic A, McLain AC, Eberth JM, Federico A, Bradshaw J, Flory K (2023). County-level prevalence estimates of ADHD in children in the United States. Ann Epidemiol.

[CR51] Zhang X, Li X, Jing Y, Fang X, Zhang X, Lei B, Yu Y (2017). Transplacental transfer of polycyclic aromatic hydrocarbons in paired samples of maternal serum, umbilical cord serum, and placenta in Shanghai, China. Environ Pollut.

