# Supplementary material for: Exposure to environmental pollutants and attention-deficit/hyperactivity disorder: an overview of systematic reviews and meta-analyses
Source: Environ Sci Pollut Res Int. 2023 Oct 12;30(52):111676–92. doi: 10.1007/s11356-023-30173-9 (PMC10643318; doi:10.1007/s11356-023-30173-9)
Supplement: Supplementary file 1 — (DOCX 299 kb) [file 11356_2023_30173_MOESM1_ESM.docx]

**SUPPLEMENTARY MATERIALS**

1. **Search strings for databases**
2. **List of excluded articles after full text reading**
3. **List of article’s funding sources**
4. **Abbreviations of pollutants**
5. **Abbreviations of ADHD assessment instruments**
6. **Assessment methods of pollutants**
7. **Scores on AMSTAR 2 items for quality assessment**

**Table 1S. Search strings for databases**

| **Database** | **Strings** |
| --- | --- |
| *Pubmed* | (((("Metals"[Mesh]) OR "Environmental Pollutants"[Mesh]) OR "Pesticides"[Mesh]) OR "Hydrocarbons"[Mesh]) AND (("Attention Deficit Disorder with Hyperactivity"[Mesh]) OR ADHD) AND humans [Mesh] |
| *Web of Science* | (ALL=(ADHD) OR ALL=(Attention Deficit Disorder with hyperactivity)) AND (ALL=(pollutants) OR ALL=(metals) OR ALL=(pesticides) OR ALL=(hydrocarbons)) AND ALL=(humans) |
| *Scopus* | ( TITLE-ABS-KEY ( metals ) OR TITLE-ABS-KEY ( pollutants ) OR TITLE-ABS-KEY ( pesticides ) OR TITLE-ABS-KEY ( hydrocarbons ) AND TITLE-ABS-KEY ( adhd ) AND TITLE-ABS-KEY ( humans ) ) |
| *Cochrane* | (Metals OR "Environmental pollutants" OR Pesticides OR hydrocarbons) AND ("Attention deficit disorder with hyperactivity" OR ADHD) |

**Table 2S. List of excluded articles after full text reading**

| **Authors, year** | **Reason for exclusion** |
| --- | --- |
| Banhela et al., 2020 | Outcome (not focused on ADHD as outcome measure) |
| Berghuis et al., 2015 | Study design (no systematic review) |
| de Araujo et al., 2016 | Study design (< 25% of included articles on ADHD) |
| de Cock et al., 2012 | Study design (no systematic review) |
| Eubig et al., 2010 | ​​Population (animal studies included) |
| Iyare, 2019 | Study design (< 25% of articles on ADHD as outcome) |
| Jurewicz & Hanke, 2011 | Outcome (not focused on ADHD as outcome measure) |
| Mostafalou & Abdollahi, 2017 | Outcome (not focused on ADHD as outcome measure) |
| Perez-Fernandez et al., 2019 | Population (animal studies included) |
| Polanska et al., 2013 | Study design (no systematic review) |
| Rodríguez-Barranco et al., 2013 | Study design (< 25% of included articles on ADHD) |
| Saikat et al., 2013 | Study design (no systematic review) |

**Table 3S. List of article’s funding sources**

| **Authors, year** | **Funding Sources** |
| --- | --- |
| Aghaei et al. (2019) | Supported by a grant (grant numbers 97-02-27-39596) from Tehran University of Medical Sciences and Health Services. |
| Donzelli et al. (2019) | No external founding |
| Forns et al. (2020) | Supported by a grant from the  European Community’s Seventh Framework Program (FP7/  2007–2013) under grant agreement Developmental Neurotoxicity  Assessment of Mixtures in Children (DENAMIC) no. 282957. |
| Goodlad et al. (2013) | Not reported |
| He et al. (2019) | Not reported |
| Kalantary et al. (2020) | Supported by the Research Center for Environmental Health Technology, Iran University of Medical Sciences, Tehran, Iran (Grant Number 98-4-99-16856). (Ethics Code: IR.IUMS.REC.1398.1037). |
| Lam et al. (2017) | Supported by John Merck Foundation, U.S. EPA (award number A124617-01), U.S. EPA (award number 83543301), National Institutes of Health/National Institute of Environmental Health Sciences (award number PO1ES022841)**.** |
| Nilsen & Tulve (2020) | Supported by an appointment to the  698 Internship/Research Participation Program at the U.S. Environmental Protection Agency (EPA), Office of Research and Development, National Exposure Research Laboratory, administered by the Oak Ridge Institute for Science and Education (ORISE) through an interagency agreement between the U.S. Department of Energy and EPA. |
| Polanska et al. (2012) | Supported by the grant PNRF-218-AI-1/07 from Norway through the Norwegian Financial Mechanism within the Polish-Norwegian Research Fund and the grant UMO-2011/01/B/NZ7/06462 from National Science Centre. |
| Praveena et al. (2020) | Not reported |
| Qu et al. (2021) | Supported by the Health Department Science Research Foundation of Hebei Province (Project No.20090057) and Innovative experiment project of Hebei Medical University (NO.USIP2020164). |
| Rivollier et al. (2019) | Not reported |
| Roth & Wilks (2014) | Supported by the Swiss Centre for Applied Human Toxcology (SCAHT) founded by the Swiss Confederation and the Universities of Geneva, Basel and Lausanne. |
| Yoshimasu et al. (2014) | Not reported |

**Table 4S. Abbreviations of pollutants**

| **Abbreviations** | **Pollutants** |
| --- | --- |
| **Air pollution** | |
| NO2 | Nitrogen Dioxide |
| SO2 | Sulfur Dioxide |
| Bz | Benzene |
| PCDD/Fs | polychlorinated dibenzo-p-furans |
| PM   - PM10 - PM2.5 - PM7 - PAH - BC/EC | Particulate matters:   - PM10 - PM2.5 - PM7 - polycyclic aromatic hydrocarbons - Black carbon/ Element carbon |
| **Phtalates (PhPl)** | |
| BPA | Biphenol-A |
| DBP | dibutyl phthalate |
| DEP | diethyl phthalate |
| DMP | dimethyl phthalate |
| BBP | butyl benzyl phthalate |
| DCHP | dicyclohexyl phthalate |
| DiNP | di-isononyl phthalate |
| DOP | Di-n-octyl phthalate |
| DEHP | Bis(2-ethylhexyl) phthalate, |
| MBP | monobutyl phthalate |
| MBzP | monobutyl phthalate |
| MECPP | mono(2-ethyl-5-carboxylpentyl) phthalate |
| MEHHP | mono(2-ethyl-5-hydroxyhexyl) phthalate |
| MEHP | monoethylhexyl phthalate |
| MEOHP | monoisopentyl phthalate |
| MEP | monoethyl phthalate |
| MMP | monomethil phtalate |
| MnBP | mono-n-butyl phthalate |
| MECPP | mono(2-ethyl-5-carboxylpentyl) phthalate |
| **Heavy metal** | |
| Pb | Lead |
| Hg | Mercury |
| Mn | Manganese |
| As | Arsenic |
| **Persistent Organic Pollutants (POPs)** | |
| PFAS   - PFOA - PFOS - PFHxS - PFNA - PFDA | Perfluoroalkyl substances   - Perfluorooctanoic acid - Perfluorooctanoic sulfonic acid - Perfluorohexanesulfonic acid - Perfluorononanoic acid - Perfluorodecanoic acid |
| PFCs | Perfluorinated compound |
| PBDEs | Polybrominated diphenyl ethers |
| Pesticides:   - DDE | Pesticides:   - Dichlorodiphenyldichloroethylene |
| PCB | Polychlorinated biphenyl |
| PCE | Perchloroethylene |
| HCB | hexachlorobenzene |
| NP | Nonylphenol |

**Table 5S. Abbreviations of ADHD assessment instruments**

| **ADHD assessment instruments** | |
| --- | --- |
| Conner’s rating scales:   1. CPRS- R 2. K-CPT-II 3. CPT and CPT-II 4. CAARS 5. CADS | Conner’s rating scales:   1. Conner’s parent rating scale-revised 2. Conners Kiddie Continuous Performance Test Second Edition 3. Conners' Continuous Performance Test II 4. Conners adult ADHD rating scales 5. Conners ADHD DSM scale |
| Manuals:   1. DSM: DSM-IV and DSM-IV-R 2. ICD | Manuals:   1. Diagnostic and Statistical Manual For Mental Disorders (fourth edition and fourth edition-revised) 2. International Classification of Diseases |
| Neuropsychological assessment:   1. ANT 2. KiTAP 3. WRAML2 4. NES:    1. SAT    2. SRTT 5. LDT | Neuropsychological assessment:   1. Child attention network test 2. Test of Attentional Performance for Children 3. Wide Range Assessment of Memory and Learning 2 4. The Neurobehavioral Evaluation System:    1. Switching Attention Test    2. Simple Reaction Time Test 5. Line Discrimination Test |
| SDQ | Strengths and Difficulties Questionnaire |
| CBCL (6-18) | Child Behavior checklist (6-18) |
| ADHD rating scale (ADHD-RS):   1. FBB-ADHS, 2. K-ARS 3. SNAP | ADHD rating scale (ADHD-RS):   1. German ADHD rating scale 2. Korean ADHD rating scale 3. Swanson, Nolan, and Pelham Version IV Scale |
| A-TAC | Autism-Tics, ADHD and other Comorbidities |
| ITSEA | Infant-Toddler Social & Emotional Assessment |
| Behavioral assessment:   1. BNBAS 2. DBDRS 3. CBSQ 4. BRS of the BSID-II 5. BASC-2 6. BASC-PRS | Behavioral assessment:   1. Brazelton Neonatal Behavioural Assessment Scale 2. Disruptive Behavioural Disorder Rating Scale 3. Behaviour Style Questionnaire–Chinese version 4. Behavior rating scale (of the The Bayley Scales of Infant Development, second edition) 5. Behavioral Assessment System for Children, Parent Rating Scale, 2nd Edition 6. Behaviour assessment system for children-parent rating scales |
| BRIEF | Behaviour rating Inventory of Executive Function |
| Temperament:   1. CTTS-R 2. CMCTQ | Temperament:   1. Revised Chinese Toddler Temperament Scale 2. Middle Childhood Temperament Questionnaire– Chinese version |
| Registres:   1. DNHR 2. NPR 3. DPCR | Registres:   1. Danish National Hospital Registry 2. Norwegian patient register 3. Danish Psychiatric Central Registry |
| BOT-2 | Bruininks-Oseretsky Test of  Motor Proficiency, 2nd edition |

**Table 6S. Assessment methods of pollutants**

| **Blood samples** | |
| --- | --- |
| - White Blood cell DNA - Cord - Cord blood - Umbilical cord white blood cells - Blood samples - Child whole blood - Maternal Blood - Anti-immune globulin exposure - Maternal negative Rh D status | |
|  |  |
|  |  |
| **Urine** | |
| - Urine samples - Urinary metabolite concentrations - BPA concentration in urine | |
| **Ambient air/air sampling** | |
| - Outdoor PAH - Indoor PAH - Concentration of the pollutants | |
| **Serum** | |
| - Maternal serum - Child serum - Cord serum | |
| **Plasma** | |
| **Breast milk concentrations** | |
| **Adducts** | |
| - Maternal adduct - Cord adduct | |
| **Other biological samples** | |
| - Analysis of teeth - Hair - X-Ray | |
|  |  |
|  |  |
| **Other assessment procedures** | |
| - Estimation in water - Interrogation - Industrial release to environments in 1998 - Distance-weighted traffic density (DWTD) - Maternal fish consumption - Vaccine adverse event reporting system (VAERS) | |
|  |  |
|  |  |
|  |  |
|  |  |
|  |  |

**Table 7S. Scores on AMSTAR 2 items for quality assessment**

| **AMSTAR-2 items** | | | | | | | | | | | | | | | | | Qualitative overall score | Qualitative rating overall confidence in the results of the review |
| --- | --- | --- | --- | --- | --- | --- | --- | --- | --- | --- | --- | --- | --- | --- | --- | --- | --- | --- |
|  | 1 | 2 | 3 | 4 | 5 | 6 | 7 | 8 | 9 | 10 | 11 | 12 | 13 | 14 | 15 | 16 |  |  |
| Aghaei et al. (2019) | + | - | + | P+ | + | - | - | + | + | - | No-MA | No-MA | + | + | No-MA | + | 8.5 | Critically low |
| Donzelli et al. (2019) | + | - | + | P+ | - | - | - | + | + | - | No-MA | No-MA | + | + | No-MA | + | 7.5 | Critically low |
| Forns et al. (2020) | + | - | + | - | - | - | - | + | - | + | + | - | - | - | - | + | 6 | Critically low |
| Goodlad et al. (2013) | - | - | + | P+ | - | - | - | - | - | - | + | - | - | + | - | - | 3.5 | Critically low |
| He et al. (2019) | + | - | + | P+ | + | + | - | + | + | - | + | + | - | + | + | + | 11.5 | Critically low |
| Kalantary et al. (2020) | + | - | + | P+ | + | - | - | + | + | - | + | + | - | + | + | - | 9.5 | Critically low |
| Lam et al. (2017) | + | + | + | + | + | + | P+ | + | + | + | No-MA | No-MA | + | + | No-MA | + | 12.5 | High |
| Nilsen & Tulve (2020) | + | - | + | P+ | - | + | - | + | + | - | + | - | - | + | + | + | 9.5 | Critically Low |
| Polanska et al. (2012) | + | - | + | P+ | - | - | - | + | - | - | No-MA | No-MA | - | - | No-MA | - | 3.5 | Critically Low |
| Praveena et al. (2020) | - | - | + | + | - | - | - | + | + | - | No-MA | No-MA | + | + | No-MA | - | 6 | Critically Low |
| Qu et al. (2021) | - | - | + | P+ | - | - | - | - | + | - | + | + | - | + | + | + | 7.5 | Critically Low |
| Rivollier et al. (2019) | + | - | + | P+ | - | - | - | - | - | - | No-MA | No-MA | - | + | No-MA | + | 4.5 | Critically Low |
| Roth & Wilks (2014) | + | P+ | + | P+ | - | - | - | + | + | - | No-MA | No-MA | + | + | No-MA | + | 8.0 | Critically Low |
| Yoshimasu et al. (2014) | + | - | + | P+ | + | + | - | + | - | - | + | + | - | + | + | + | 10.5 | Critically Low |

Notes: High=No or one non-critical weakness: the systematic review provides an accurate and comprehensive summary of the results of the available studies that address the question of interest; Moderate=More than one non-critical weakness*: the systematic review has more than one weakness but no critical flaws. It may provide an accurate summary of the results of the available studies that were included in the review; Low= One critical flaw with or without non-critical weaknesses: the review has a critical flaw and may not provide an accurate and comprehensive summary of the available studies that address the question of interest; Critically low= More than one critical flaw with or without non-critical weaknesses: the review has more than one critical flaw and should not be relied on to provide an accurate and comprehensive summary of the available studies.
